# Are hypothalamic- pituitary (HP) axis deficiencies after whole brain radiotherapy (WBRT) of relevance for adult cancer patients? – a systematic review of the literature

**DOI:** 10.1186/s12885-019-6431-5

**Published:** 2019-12-12

**Authors:** P. Mehta, F. B. Fahlbusch, D. Rades, S. M. Schmid, J. Gebauer, S. Janssen

**Affiliations:** 1 Department of Radiation Oncology, University of Lübeck, Ratzeburger Allee 160, 23538 Lübeck, Germany; 2 Department of Pediatrics and Adolescent Medicine, Friedrich-Alexander-University of Erlangen-Nürnberg, Erlangen, Germany; 3 Department of Endocrinology, University of Lübeck, Lübeck, Germany; 4 German Center for Diabetes Research (DZD), Neuherberg, Germany; 5 Private Practice of Radiation Oncology, Hannover, Germany

**Keywords:** Whole brain radiotherapy, Endocrine deficiencies, Hypothalamus, Pituitary, Hypopituitarism

## Abstract

**Background:**

Cranial radiotherapy (cRT) can induce hormonal deficiencies as a consequence of significant doses to the hypothalamic-pituitary (HP) axis. In contrast to profound endocrinological follow-up data from survivors of childhood cancer treated with cRT, little knowledge exists for adult cancer patients.

**Methods:**

A systematic search of the literature was conducted using the PubMed database and the Cochrane library offering the basis for our debate of the relevance of HP axis impairment after cRT in adult cancer patients. Against the background of potential relevance for patients receiving whole brain radiotherapy (WBRT), a particular focus was set on the temporal onset of hypopituitarism and the radiation dose to the HP axis.

**Results:**

Twenty-eight original papers with a total of 1728 patients met the inclusion criteria. Radiation doses to the HP area ranged from 4 to 97 Gray (Gy). Hypopituitarism incidences ranged from 20 to 93% for adult patients with nasopharyngeal cancer or non-pituitary brain tumors. No study focused particularly on hypopituitarism after WBRT. The onset of hypopituitarism occurred as early as within the first year following cRT (range: 3 months to 25.6 years). However, since most studies started follow-up evaluation only several years after cRT, early onset of hypopituitarism might have gone unnoticed.

**Conclusion:**

Hypopituitarism occurs frequently after cRT in adult cancer patients. Despite the general conception that it develops only after several years, onset of endocrine sequelae can occur within the first year after cRT without a clear threshold. This finding is worth debating particularly in respect of treatment options for patients with brain metastases and favorable survival prognoses.

## Background

Endocrine long-term complications are common in childhood cancer survivors [1–6]. After cranial radiotherapy (cRT), multiple endocrine functions can be affected [7, 8]. In a large retrospective study with a cohort of 748 childhood cancer survivors treated with cRT, the estimated point prevalence for growth hormone (GH) deficiency was 46.5%, 10.8% for thyroid-stimulating (TSH) hormone deficiency and 4% for adrenocorticotropic hormone deficiency (ACTH) [9], respectively. As young age at the time of radiation represents a risk factor for the development of endocrine deficiencies after cRT [10], the prevalence of cRT-induced hypothalamic-pituitary (HP) axis-related sequelae in adult patients may vary.

In contrast to comprehensive data on childhood cancer patients, information on hormonal impairment after cRT in adults is scarce. Reviewing eighteen studies with a total of 813 adult cancer patients treated with cRT (nasopharyngeal cancer and non-pituitary brain tumors) in 2011, Appelman-Dijkstra et al. were able to show that the prevalence of cRT-induced hypopituitarism was of clinical relevance and argued for a structured periodical endocrine follow-up of these adults [11].

We aimed to update the existing knowledge database on HP axis dysfunction after cRT in adult cancer patients (non-pituitary brain tumors and nasopharyngeal cancer) by including current literature for further debate. We were able to gather more detailed information on the course of HP axis impairment after WBRT in adults, enabling the discussion of the following clinical questions in particular: i) What is the current evidence on HP axis dysfunction after WBRT in literature? ii) Are WBRT doses within the range of potential harm? iii) Can we neglect the impact of cRT-related hormonal deficiency in patients with brain metastases and limited life expectancy as a typically late manifesting side effect?

## Methods

**Fig. 1:**
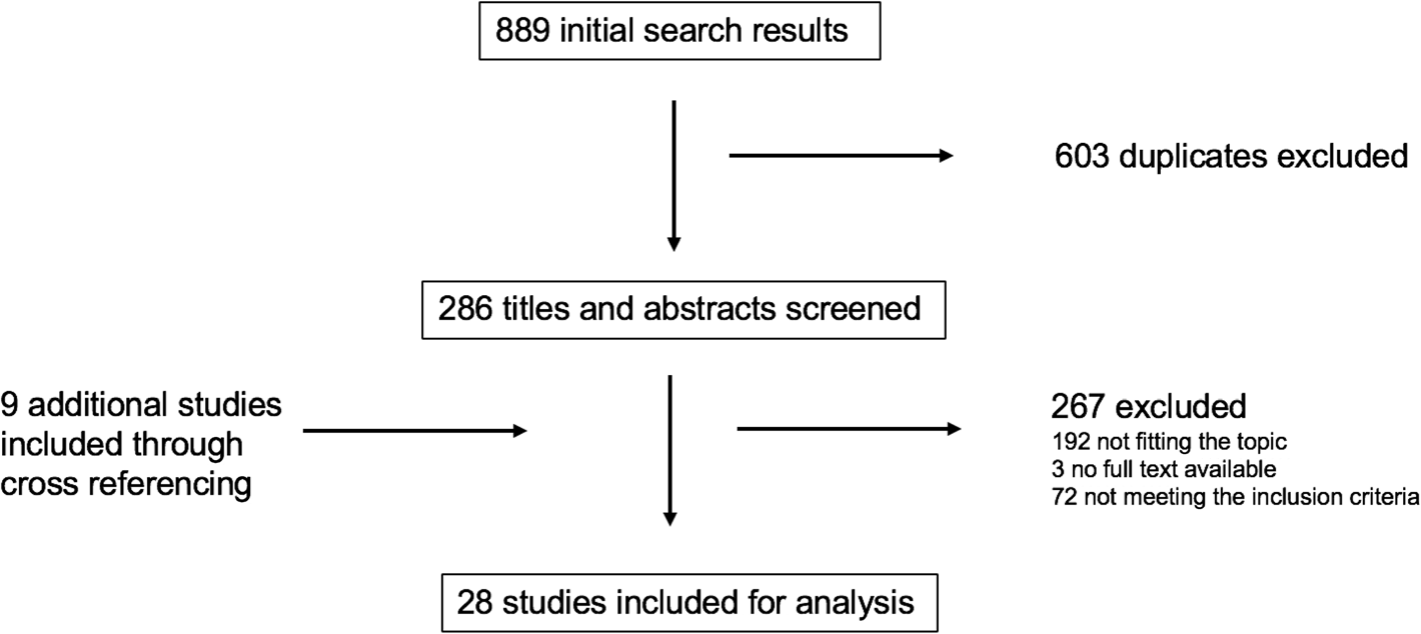
PRISMA flow chart summarizing the selection process

On April 25th, 2019 the PubMed database and the Cochrane library were searched for the following terms: “cranial radiotherapy” OR “cranial irradiation” AND “hypopituitarism” OR “pituitary deficiency” OR “hormonal changes” OR “hormonal impairment” OR “hormonal deficiency”. Only studies written in English on adult cancer patients (≥18 years, non-pituitary brain tumors, head and neck cancers and brain metastases) with information on endocrine function after cRT were considered. The initial search was supplemented with manual searches of the reference lists and cross-referencing. A PRISMA flow chart [12] summarizes the selection process (Fig. 1).

## Results

The initial search returned 889 citations, with 286 remaining after removing duplicates and limiting the results to adult cancer patients (Fig. 1). After screening titles and abstracts, 28 original full papers with a total of 1728 patients were included.

Search results could mainly be assigned to the tumor entities nasopharynx carcinoma/ skull base tumors (15 studies) and non-pituitary brain tumors (13 studies). No study with a focus on endocrine assessment after WBRT in adult cancer patients was found.

The overall prevalence of pituitary dysfunction ranged from 20 to 93% (GH: 19–100%, ACTH: 4–73%, TSH: 4–70%, LH/FSH: 0–55%, PRL: 7–100%). The time interval from cRT to endocrine assessment ranged from 3 months to 25.6 years. In 13 studies, the median start of follow-up exceeded three years. The total radiation dose to the pituitary gland/hypothalamus ranged from 4 to 97 Gy. Eight studies did not supply estimated doses to the HP axis. Table 1 summarizes the search results.

**Table 1:** Studies with data on pituitary hormonal impairment after cranial radiotherapy in adult cancer patients

Author	Patients	Age (years)	Tumor entity	RT dose (Gy)	Time since RT	Pituitary hormonal impairment (axis)					
						Any deficiency	GH deficiency	ACTH deficiency	TSH deficiency	LH/FSH deficiency	PRL (hyperprolactinemia)
Lamba 2019 [13]	74	NR	Meningeoma	NR	Follow up: mean: 43 months Mean time to develop hormone deficiency: 11–32 months		19% (14/74)	24% (18/74)	24% (18/74)	10% (7/74)	NR
Handisurya 2018 (prospective) [14]	436	52 (19–83)	Brain	54–60 Gy No statement on dose to the pituitary/hypothalamus	Baseline 3 months after RT 6 months after RT 1 years after RT 2 years after RT 3 years after RT > 3 years after RT		NR	NR	21% 16.8% 11.3% 10.2% 1.4% 5.7% 10.9%	0% / 0%* 7.1% / 0%* 32.7% / 0%* 35.3% / 0%* 41.7% / 0%* 52.2% / 0%* 24% / 0%* LH: always 0%	20% / 11%* 2% / 18.8%* 1.3% / 6.8%* 3.1% / 10.3%* 22.2% / 0%* 55.6% / 4.3%* 26% / 42.1%*
Kyriakakis 2016 [15]	107	40 ± 13.1	Brain	54Gy, estimated dose to the HP axis 35.9 ± 15.5Gy**	8 years 5.3–11 years	88%	86.9%	23.4%	11.2%	34.6%	15%
Ipekci 2014 [16]	30	45.2 ± 9.8	Nasopharyngeal	Mean dose to the pituitary: 46Gy (23–66) Mean dose to the hypothalamus: 10Gy (4–41)	10–133 months	93% (28/30)	77% (23/30)	73% (22/30)	27% (8/30)	NR	43% (13/30)
Ratnasingam 2014 [17]	50	57 ± 12.2	Nasopharyngeal	No statement on dose to the pituitary/hypothalamus	Median: 8 years (3–21)	82% (41/50)	78% (39/50)	40% (20/50)	4% (4/50)	22% (11/50)	30% (15/50)
Seland 2014 [18]	140	42.5 (15–76)	Head and neck	Pituitary: 13Gy (0–68)	16 years (5–29)	73% (102/140)	25% (32/140)	34% (45/140)	34% (45/140)	55%	NR
Appelman-Dijstra 2014 [19]	80	47.5 (18.6–89.7)	Brain	Pituitary: 56.27 (40–70)	2 years after RT 5 years after RT 10 years after RT 15 years after RT	21% (17/80) 47% (23/49) 60% (27/45) 89% (31/35)	33% (27/80)	31% (25/80)	14% (11/80)	25% (20/80)	21% (17/80)
Madischi 2011 [20]	26	38.5 (33–47)	Brain	Mean dose to the pituitary and hypothalamus: 41.8 Gy (30.7–49.8)	< 32 months (median: 27 months)	38% (10/26)	29% (7/26)	22% (6/26)	14% (4/26)	4% (1/26)	NR
Snyders 2009 [21]	76, 21 with endocrine evaluation	56 (28–74)	Sinunasal	44-66Gy, estimated dose to the pituitary: 51–56, estimated dose to the hypothalamus: 44-52Gy	107 months (11–253)	62% (13/21)	24% (5/21)	19% (4/21)	14% (3/21)	19% (4/21)	10% (2/21)
Bhadare 2008 [22]	312, 112 with endocrine evaluation	NR	Nasopharyngeal	40-70Gy	63 months (6–365)	60% (67/112)	36% (16/44)	32% (14/44)	70% (31/44)	27% (12/44)	15% (10/68)
Schneider 2006 [23]	68, 44 with endocrine evaluation	20–79	Brain	NR	NR	38% (17/44)	28% (12/44)	19% (8/44)	18% (7/44)	29.5% (13/44)	7% (3/44)
Agha 2005 [24]	56	39.3 ± 11.9	Brain	Estimated dose to the pituitary: 54Gy (4–97)	6 years	41% (23/56)	32% (18/56)	21% (12/56)	9% (5/56)	27% (15/56)	32% (18/56)
Johannesen 2003 [25]	33, 25 with endocrine evaluation	38 (14–68)	Brain	54 Gy (45–59)	13.1 years (6–25.6)	64% (16/25)	NR	4% (1/25)	56% (14/25)	16% (4/25)	0% (0/25)
Popovic 2002 [26]	22 6 > 18y	NR	Brain	56 Gy, estimated dose to the pituitary: 25-30Gy	7.6 ± 0.7 years (2–3)	67% (4/6)	67% (4/6)	NR	NR	NR	NR
Pai 2001 [27]	107	41.2 (17–75)	Chordoma/ chondrosarcoma	Estimated dose to pituitary and hypothalamus: < 50-70Gy	5.5 years	87%	NR	19%	30%	29%	72%
Arlt 1997 [28]	31	26–66	Brain	Mean dose pituitary: 51.1Gy (±12.1) mean dose hypothalamus: 57Gy (±7.8)	1.5–11 years	77% (24/31)				NR	29% (9/31)
Taphoorn 1995 [29]	13	24–66	Brain	45–61 Gy, mean pituitary dose: 36.1 Gy (0–50)	3 years (1–11.5)	77% (10/13)	31% (4/13)	62% (8/13)	0% (0/13)	15% (2/13)	23% (3/13)
Constine 1993 [30]	32	6–65	Brain	39.6–70.2Gy	2–13 years	91% (28/32)	NR	NR	NR	NR	NR
Lam 1991 [31]	20	43.7 ± 8.4 (male) 36.8 ± 9 (female)	Nasopharyngeal	Estimated dose to the pituitary 62Gy, estimated dose to the hypothalamus: 40Gy	60 months	75% (15/20)	55% (11/20)	25% (5/20)	15% (3/20)	35% (7/20)	30% (6/20)
Woo 1988 [32]	11	33–64	Nasopharyngeal	Estimated dose to pituitary 62-67Gy, estimated dose to the hypothalamus: 41-45Gy	72–240 months	82% (9/11)	90% (9/10)	18% (2/11)	45% (5/11)	55% (6/11)	27% (3/11)
Samaan 1987 [33]	166	47 (6–80)	Nasopharyngeal and paranasal sinus	Estimated dose to the pituitary: 57 (4–75), estimated dose to the hypothalamus: 50 (11–75)	12–312 months	75% (124/166)	75% (124/166)	18% (30/166)	20% (33/166)	20% LH (33/166) 35% FSH (58/166)	36% (60/166)
Lam 1987 [34]	32	27–50	Nasopharyngeal	46–60	60–204 months	25% (8/32)	19% (6/32)	6% (2/32)	13% (4/32)	16% (5/32)	19% (6/32)
Mechanik 1986 [35]	15	22–59	Brain	40–50 to whole brain	2–9 years	93% (14/15)	NR	NR	14.3% (1/7)	NR	100% (9/9)
Lam 1986 [36]	8	27–52	Nasopharyngeal	46–61, estimated to the pituitary 55–67	> 60 months	100% (8/8)	100% (8/8)	50% (4/8)	50% (4/8)	25% (2/8)	88% (7/8)
Huang 1979 (prospective) [37]	37	16–65	Nasopharyngeal	estimated dose to pituitary/hypothalamus: 46–56 Gy	Before RT 6 months after RT 1 years after RT 2 years after RT	NR	8.1% 18.9% 18.9% 21.6% NS	46% 73% 83.8% 83.8% p = < 0.01	0% 21.6% 27% 37.8% p = < 0.01	10.8% / 48.6% 56.8% / 83.8% 51.4% / 83.8% 56.8% / 81.1% (LH/FSH) *p* < 0.01	NR
Harrop 1976 [38]	17 8 < 18y	8 < 18	Brain	40–52	6 years (1–15)	62.5% (5/8)	50% (4/8)	12.5% (1/8)	12.5% (1/8)	37.5% (3/8)	NR
Rosenthal 1976 [39]	6	35–66	Nasopharyngeal	55–65	12–96 months	67% (4/6)	100% (2/2)	50% (1/2)	67% (4/6)	NR	NR
Samaan 1975 [40]	10	26–55	Nasopharyngeal	Estimated dose to pituitary: 50–83	60–240 months	100% (10/10)	60% (6/10)	50% (5/10)	40% (4/10)	30% (3/10)	50% (5/10)

Abbreviations: *GH* growth hormone, *ACTH* adrenocorticotropic hormone, *TSH* thyroid-stimulating hormone, *LH* luteinizing hormone, *FSH* follicle-stimulating hormone, *PRL* prolactin, *Gy* Gray, *RT* radiation therapy, *NR* Not reported; * younger and older than 50 years, ** subgroup analysis of the study group (Kyriakakis et al.)

## Discussion

The review of 28 original articles showed that hypopituitarism occurs frequently in adult patients after cRT for nasopharyngeal cancer and non-pituitary brain tumors. The fact that not a single article was focused on hypopituitarism after WBRT in particular highlights the general need for our review of HP axis deficiencies after cRT to improve extrapolation of the results for WBRT patients.

In 2011, Appelman-Dijkstra et al. published a review and meta-analysis on pituitary dysfunction in adult patients after cRT including 18 studies with a total of 813 patients [11]. The authors found hypopituitarism prevalent and concluded that all patients treated with cRT should undergo structured periodical assessment of pituitary functions [11]. In the meantime, ten additional articles published including 915 new patients, justifying an update of the pre-existing database regarding the current literature. Our analysis additionally contributes to the debate of whether the onset of pituitary dysfunction and radiation dose leading to pituitary dysfunction could be of relevance for patients treated with WBRT.

### Manifestation of cRT-induced endocrine deficiencies - potential relevance for patients with brain metastases and limited life expectancy?

Brain metastases are the most common brain tumors in adults, occurring in approximately 10–30% of adult cancer patients [41]. While WBRT remains a primary treatment modality for many patients with high intracranial tumor burden, there has been a paradigm shift from WBRT to stereotactic small volume high precision RT (stereotactic surgery (SRS)) in patients with a limited number a brain metastases. However, there is no general consensus defining the cut-off for the number of lesions. Randomized studies support the use of SRS in 1–3 lesions [42–44] or up to 10 metastases [45]. Still, WBRT (preferably with hippocampal sparing technique) remains an option for patients with more than a “limited number” of brain metastases.

In general, life expectancy of patients with brain metastases is limited [41]. Recursive partioning analysis (RPA) of prognostic factors in Radiation Treatment Oncology Group (RTOG) brain metastases trials suggested three prognostic classes with median survival rates from 2.3 months to 7.1 months [46]. More recently published prognostic scores, not only based on performance status and age, showed a more differentiated range of life expectancies in patients with brain metastases [47–49]. According to the scoring system established by Rades et al., the group with the best prognosis showed a 1-year survival rate of 49% [48]. With the disease specific Graded Prognostic Assessment (GPA) score introduced by Spertudo et al., median survival of up to 18.7 months can be reached depending on the primary tumor site [47].

All together, there are subgroups of patients with brain metastases that live significantly longer than the average. Apart from patients presenting with brain metastases, a prophylactic brain irradiation (PCI) was shown to be beneficial in patients with small cell lung cancer (SCLC) without evidence of intracranial disease [50, 51]. In cases of limited disease and complete remission after chemotherapy, survival rates sum up to approximately 60 and 35% after 1-year and 2-years, respectively [50].

While the mean time to follow-up was longer than two years in the majority of analyzed studies, most studies also showed that hypopituitarism can already occur in the first year after treatment [13, 14, 16, 19–22, 29, 31, 33, 37]. In particular, Lamba et al. reported all hormonal deficiencies in their study after a mean time of 11–32 months [13]. All hypothalamic-pituitary insufficiencies occurred within 32 months after cRT in the study of Madaschi et al. [20]. Handisurya et al. showed in their longitudinal study that low levels of thyroid and sexual hormones occur in a significant proportion of patients within the first months after initiation of therapy [14]. The authors assumed that early hormonal impairment might be a coping mechanism of the body that contributes to the increased fatigue and weakness reported by many patients during or after cRT. While this toxicity is mostly interpreted as acute toxicity of cRT and/or side effect of steroid medication (used for symptomatic treatment of vasogenic edema), it is plausible that hormonal deficiencies, especially of anabolic hormones, and the lack of sexual functioning might contribute to an acute illness syndrome in this period [52]. The incidence of hormonal deficiencies within six months after treatment start has not yet been reported and a potential benefit of hormonal replacement therapies has not yet been studied, as most studies started their endocrine follow-up in the second year after cRT or later [14].

### Are doses being used for WBRT within the range of potential harm to the HP axis?

Multiple dose regimes are common for WBRT ranging from 5 × 4 = 20 Gy, 10 × 3 = 30 Gy, 15 × 2.5 = 37.5 Gy and 20 × 2 = 40 Gy [53–56]. As the irradiated volume encompasses the entire cerebrum, the HP axis is always being treated with the total description dose. Several studies showed a dose-dependent effect on the development of hypopituitarism [10, 13, 22, 24, 56, 57]. So far, however, no threshold dose for hormonal impairment has been established. In the studies reviewed, doses to the pituitary and the hypothalamus ranged between 4 and 97 Gy. In half of the studies, a minimum dose to the HP area of less than 40 Gy was mentioned. Kyriakakis et al. suggested a threshold dose above 30 Gy to the HP area for long term endocrine surveillance [56]. This is thoroughly within the dose range of a WBRT.

The vast majority of patients in the analyzed studies was treated with conventionally fractionated RT (1.8–2.0 Gy per fraction). In case of WBRT, a hypo-fractionated regime is often used (e.g. 10 × 3 = 30 Gy). Assuming an α/ß ratio of 3 for normal brain tissue, this biologically equals significantly higher doses. The lower the dose per fraction, the greater the sparing of late-reaction tissue [58]. The HP axis behaves as a late-reacting tissue. A dose per fraction of more than 2 Gy administered over a shorter time period has a more severe effect on late-reacting tissues such as myelinated neurons and blood vessels [7, 59]. More profound cRT-induced hypopituitarism has been demonstrated with a dose per fraction greater than 2 Gy in older series [60]. So far, however, no direct comparative data exists clearly supporting this hypothesis.

Moreover, modern cancer treatment approaches like immunotherapy carry the risk of additional autoimmune hypophysitis [61]. To the best of our knowledge, no data exists on a synergistic risk of HP axis impairment with cRT and immunotherapy. In expectation of a more frequent use of immunotherapy, this topic might become of importance in the future.

Certain limitations might limit the scope of our analysis: Data on onset of hypopituitarism and estimated dose to the HP area were not completely reported by all included studies. For instance, eight studies did not supply estimated doses to the HP axis at all. The time interval from cRT to assessment of endocrine deficiencies was often documented as a range. In those cases, it is not known, how many patients developed a deficiency within the first two years and how many patients after a longer follow-up period. These ranges were included in our overview (Table 1). Moreover, our analysis revealed a large variability in the prevalence of pituitary dysfunction (range 20–93%) and the total radiation dose to the HP axis (range 4–97 Gy) complicating interpretation. In addition, endocrine function testing varied substantially in the included studies. Also, as described by Merchant et al. in pediatric patients, pre-irradiation endocrinopathies might lead to a potential overestimation of the incidence of radiation-induced endocrinopathy [62].

In lack of sufficient data, our conclusions were drawn from subpopulations, retrospective and heterogenous patient groups. Thus, while our hypotheses fall short of translating into patient care strategies, it clearly demonstrates the current need for future studies regarding dose constraints in order to improve care delivery, such as a sparing approach in WBRT and/or endocrine follow-up. Such trials should especially emphasize on rigorous data acquisition including relevant dose constraints to the HP axis so that ongoing prospective efforts can better address these issues.

Nonetheless, we believe that the extrapolation of published data on hypopituitarism in adult cancer patients with nasopharyngeal cancer and brain tumors should suffice to justify early routine endocrinological follow-up (within the first year after cRT) for patients with brain metastases and a favorable prognosis receiving WBRT or in patients with SCLC in need for a PCI. In order to prevent hypopituitarism after WBRT, a sparing approach of the HP axis might be an option and appears technically feasible (unpublished data of our study group).

## Conclusion

Hypopituitarism after cRT in adult cancer patients occurs frequently, is dose dependent and can evolve within the first year after RT. A search of the current literature returned no data on hypopituitarism after WBRT in particular. Especially when treating a subgroup of patients with a favorable prognosis with WBRT one should consider the presented follow-up data from patients receiving cRT of nasopharyngeal cancer and brain tumors, as hypopituitarism has been described to occur within the dose range of WBRT (20–40 Gy) without a dose threshold. On the basis of the current literature, our debate further highlights the need for endocrinological evaluation following cRT and should encourage the initiation of further studies regarding a sparing approach of the HP area during WBRT.

## Data Availability

Not applicable, entire data is shown within the manuscript / tables.

## Abbreviations

ACTH: Adrenocorticotropin hormone
cRT: cranial radiotherapy
GH: Growth hormone
GPA: Graded prognostic assessment
Gy: Gray
HP: Hypothalamic-pituitary
LF/FSH: Luteinizing hormone/follicle-stimulating hormone
PCI: Prophylactic cranial irradiation
PRL: Prolactin
RPA: Recursive partioning analysis
RTOG: Radiation Therapy Oncology Group
SCLC: Small cell lung cancer
SRS: Stereotactic radiosurgery
TSH: Thyroid-stimulating hormone
WBRT: Whole brain radiotherapy

## References

[1] Gebauer J, Higham C, Langer T, Denzer C, Brabant G. Long-term Endocrine and Metabolic consequences of Cancer Treatment: A Systematic Review. Endocr Rev. 2018 Nov;23. 10.1210/er.2018-00092 30476004

[2] Merchant TE, Goloubeva O, Pritchard DL, Gaber MW, Xiong X, Danish RK, Lustig RH (2002). Radiation dose-volume effects on growth hormone secretion. Int J Radiat Oncol Biol Phys.

[3] Hua C, Wu S, Chemaitilly W, Lukose RC, Merchant TE (2012). Predicting the probability of abnormal stimulated growth hormone response in children after radiotherapy for brain tumors. Int J Radiat Oncol Biol Phys.

[4] Merchant TE, Rose SR, Bosley C, Wu S, Xiong X, Lustig RH (2011). Growth hormone secretion after conformal radiation therapy in pediatric patients with localized brain tumors. J Clin Oncol.

[5] Merchant TE, Conklin HM, Wu S, Lustig RH, Xiong X (2009). Late effects of conformal radiation therapy for pediatric patients with low-grade glioma: prospective evaluation of cognitive, endocrine, and hearing deficits. J Clin Oncol.

[6] Laughton SJ, Merchant TE, Sklar CA, Kun LE, Fouladi M, Broniscer A, Morris EB, Sanders RP, Krasin MJ, Shelso J, Xiong Z, Wallace D, Gajjar A (2008). Endocrine outcomes for children with embryonal brain tumors after risk-adapted craniospinal and conformal primary-site irradiation and high-dose chemotherapy with stem-cell rescue on the SJMB-96 trial. J Clin Oncol.

[7] Pekic S, Miljic D, Popovic V. Hypopituitarism Following Cranial Radiotherapy. In: Feingold KR, Anawalt B, Boyce A, Chrousos G, Dungan K, Grossman A, Hershman JM, Kaltsas G, Koch C, Kopp P, Korbonits M, McLachlan R, Morley JE, New M, Perreault L, Purnell J, Rebar R, Singer F, Trence DL, Vinik A, Wilson DP, editors. Endotext [Internet]. South Dartmouth (MA): MDText.com, Inc.; 2000-. 2018 Oct 1.

[8] Darzy KH, Shalet SM (2009). Hypopituitarism following radiotherapy. Pituitary.

[9] Chemaitilly W, Li Z, Huang S, Ness KK, Clark KL, Green DM, Barnes N, Armstrong GT, Krasin MJ, Srivastava DK, Pui CH, Merchant TE, Kun LE, Gajjar A, Hudson MM, Robison LL, Sklar CA (2015). Anterior hypopituitarism in adult survivors of childhood cancers treated with cranial radiotherapy: a report from the St Jude Lifetime Cohort study. J Clin Oncol.

[10] Vatner RE, Niemierko A, Misra M, Weyman EA, Goebel CP, Ebb DH, Jones RM, Huang MS, Mahajan A, Grosshans DR, Paulino AC, Stanley T, MacDonald SM, Tarbell NJ, Yock TI (2018). Endocrine deficiency as a function of radiation dose to the hypothalamus and pituitary in pediatric and young adult patients with brain tumors. J Clin Oncol.

[11] Appelman-Dijkstra NM, Kokshoorn NE, Dekkers OM, Neelis KJ, Biermasz NR, Romijn JA, Smit JW, Pereira AM (2011). Pituitary dysfunction in adult patients after cranial radiotherapy: systematic review and meta-analysis. J Clin Endocrinol Metab.

[12] Liberati A, Altman DG, Tetzlaff J, Mulrow C, Gøtzsche PC, Ioannidis JP, Clarke M, Devereaux PJ, Kleijnen J, Moher D (2009). The PRISMA statement for reporting systematic reviews and meta-analyses of studies that evaluate health care interventions: explanation and elaboration. PLoS Med.

[13] Lamba N, Bussiere MR, Niemierko A, Abedi P, Fullerton BC, Loeffler JS, Oh KS, Nachtigall LB, Shih HA. Hypopituitarism After Cranial Irradiation for Meningiomas: A Single-Institution Experience. Pract Radiat Oncol. 2019 Feb 4. pii: S1879–8500(19)30043–30048. 10.1016/j.prro.2019.01.009 30731274

[14] Handisurya A, Rumpold T, Caucig-Lütgendorf C, Flechl B, Preusser M, Ilhan-Mutlu A, Dieckmann K, Widhalm G, Grisold A, Wöhrer A, Hainfellner J, Ristl R, Kurz C, Marosi C, Gessl A, Hassler M (2019). Are hypothyroidism and hypogonadism clinically relevant in patients with malignant gliomas? A longitudinal trial in patients with glioma. Radiother Oncol.

[15] Kyriakakis N, Lynch J, Orme SM, Gerrard G, Hatfield P, Loughrey C, Short SC, Murray RD (2016). Pituitary dysfunction following cranial radiotherapy for adult-onset nonpituitary brain tumours. Clin Endocrinol.

[16] Ipekci SH, Cakir M, Kiyici A, Koc O, Artac M (2015). Radiotherapy-induced hypopituitarism in nasopharyngeal carcinoma: the tip of an iceberg. Exp Clin Endocrinol Diabetes.

[17] Ratnasingam J, Karim N, Paramasivam SS, Ibrahim L, Lim LL, Tan AT, Vethakkan SR, Jalaludin A, Chan SP (2015). Hypothalamic pituitary dysfunction amongst nasopharyngeal cancer survivors. Pituitary..

[18] Seland M, Bjøro T, Furre T, Schreiner T, Bollerslev J, Fosså SD, Loge JH, Holte H, Kiserud CE (2015). Hormonal dysfunction is frequent in cancer survivors treated with radiotherapy to the head and neck region. J Cancer Surviv.

[19] Appelman-Dijkstra NM, Malgo F, Neelis KJ, Coremans I, Biermasz NR, Pereira AM (2014). Pituitary dysfunction in adult patients after cranial irradiation for head and nasopharyngeal tumours. Radiother Oncol.

[20] Madaschi S, Fiorino C, Losa M, Lanzi R, Mazza E, Motta M, Perna L, Brioschi E, Scavini M, Reni M (2011). Time course of hypothalamic-pituitary deficiency in adults receiving cranial radiotherapy for primary extrasellar brain tumors. Radiother Oncol.

[21] Snyers A, Janssens GO, Twickler MB, Hermus AR, Takes RP, Kappelle AC, Merkx MA, Dirix P, Kaanders JH. Malignant tumors of the nasal cavity and paranasal sinuses: long-term outcome and morbidity with emphasis on hypothalamic-pituitary deficiency. Int J Radiat Oncol Biol Phys 2009 Apr 1;73(5):1343–1351. doi: 10.1016/j.ijrobp.2008.07.040. Epub 2008 Oct 27. 10.1016/j.ijrobp.2008.07.040 18963535

[22] Bhandare N, Kennedy L, Malyapa RS, Morris CG, Mendenhall WM (2008). Hypopituitarism after radiotherapy for extracranial head and neck cancers. Head Neck.

[23] Schneider HJ, Rovere S, Corneli G, Croce CG, Gasco V, Rudà R, Grottoli S, Stalla GK, Soffietti R, Ghigo E, Aimaretti G (2006). Endocrine dysfunction in patients operated on for non-pituitary intracranial tumors. Eur J Endocrinol.

[24] Agha A, Sherlock M, Brennan S, O'Connor SA, O'Sullivan E, Rogers B, Faul C, Rawluk D, Tormey W, Thompson CJ Hypothalamic-pituitary dysfunction after irradiation of nonpituitary brain tumors in adults. J Clin Endocrinol Metab 2005 Dec;90(12):6355–6360. Epub 2005 Sep 6. 10.1210/jc.2005-1525 16144946

[25] Johannesen TB, Lien HH, Hole KH, Lote K (2003). Radiological and clinical assessment of long-term brain tumour survivors after radiotherapy. Radiother Oncol.

[26] Popovic V, Pekic S, Golubicic I, Doknic M, Dieguez C, Casanueva FF (2002). The impact of cranial irradiation on GH responsiveness to GHRH plus GH-releasing peptide-6. J Clin Endocrinol Metab.

[27] Pai HH, Thornton A, Katznelson L, Finkelstein DM, Adams JA, Fullerton BC, Loeffler JS, Leibsch NJ, Klibanski A, Munzenrider JE (2001). Hypothalamic/pituitary function following high-dose conformal radiotherapy to the base of skull: demonstration of a dose-effect relationship using dose-volume histogram analysis. Int J Radiat Oncol Biol Phys.

[28] Arlt W, Hove U, Müller B, Reincke M, Berweiler U, Schwab F, Allolio B (1997). Frequent and frequently overlooked: treatment-induced endocrine dysfunction in adult long-term survivors of primary brain tumors. Neurology..

[29] Taphoorn MJ, Heimans JJ, van der Veen EA, Karim AB (1995). Endocrine functions in long-term survivors of low-grade supratentorial glioma treated with radiation therapy. J Neuro-Oncol.

[30] Constine LS, Woolf PD, Cann D, Mick G, McCormick K, Raubertas RF, Rubin P (1993). Hypothalamic-pituitary dysfunction after radiation for brain tumors. N Engl J Med.

[31] Lam KS, Tse VK, Wang C, Yeung RT, Ho JH (1991). Effects of cranial irradiation on hypothalamic-pituitary function--a 5-year longitudinal study in patients with nasopharyngeal carcinoma. Q J Med.

[32] Woo E, Lam K, Yu YL, Ma J, Wang C, Yeung RT (1988). Temporal lobe and hypothalamic-pituitary dysfunctions after radiotherapy for nasopharyngeal carcinoma: a distinct clinical syndrome. J Neurol Neurosurg Psychiatry.

[33] Samaan NA, Schultz PN, Yang KP, Vassilopoulou-Sellin R, Maor MH, Cangir A, Goepfert H (1987). Endocrine complications after radiotherapy for tumors of the head and neck. J Lab Clin Med.

[34] Lam KS, Ho JH, Lee AW, Tse VK, Chan PK, Wang C, Ma JT, Yeung RT (1987). Symptomatic hypothalamic-pituitary dysfunction in nasopharyngeal carcinoma patients following radiation therapy: a retrospective study. Int J Radiat Oncol Biol Phys.

[35] Mechanick JI, Hochberg FH, LaRocque A (1986). Hypothalamic dysfunction following whole-brain irradiation. J Neurosurg.

[36] Lam KS, Wang C, Yeung RT, Ma JT, Ho JH, Tse VK, Ling N (1986). Hypothalamic hypopituitarism following cranial irradiation for nasopharyngeal carcinoma. Clin Endocrinol.

[37] Huang TS, Huang SC, Hsu MM (1994). A prospective study of hypothalamus pituitary function after cranial irradiation with or without radiosensitizing chemotherapy. J Endocrinol Investig.

[38] Harrop JS, Davies TJ, Capra LG, Marks V (1976). Hypothalamic-pituitary function following successful treatment of intracranial tumours. Clin Endocrinol.

[39] Rosenthal MB, Goldfine ID (1976). Primary and secondary hypothyroidism in nasopharyngeal carcinoma. JAMA.

[40] Samaan NA, Vieto R, Schultz PN, Maor M, Meoz RT, Sampiere VA, Cangir A, Ried HL, Jesse RH (1982). Hypothalamic, pituitary and thyroid dysfunction after radiotherapy to the head and neck. Int J Radiat Oncol Biol Phys.

[41] Khuntia D, Brown P, Li J, Mehta MP (2006). Whole-brain radiotherapy in the management of brain metastasis. J Clin Oncol.

[42] Andrews DW, Scott CB, Sperduto PW, Flanders AE, Gaspar LE, Schell MC, Werner-Wasik M, Demas W, Ryu J, Bahary JP, Souhami L, Rotman M, Mehta MP, Curran WJ (2004). Whole brain radiation therapy with or without stereotactic radiosurgery boost for patients with one to three brain metastases: phase III results of the RTOG 9508 randomised trial. Lancet.

[43] Aoyama H, Shirato H, Tago M, Nakagawa K, Toyoda T, Hatano K, Kenjyo M, Oya N, Hirota S, Shioura H, Kunieda E, Inomata T, Hayakawa K, Katoh N, Kobashi G (2006). Stereotactic radiosurgery plus whole-brain radiation therapy vs stereotactic radiosurgery alone for treatment of brain metastases: a randomized controlled trial. JAMA.

[44] Kocher M, Soffietti R, Abacioglu U, Villà S, Fauchon F, Baumert BG, Fariselli L, Tzuk-Shina T, Kortmann RD, Carrie C, Ben Hassel M, Kouri M, Valeinis E, van den Berge D, Collette S, Collette L, Mueller RP (2011). Adjuvant whole-brain radiotherapy versus observation after radiosurgery or surgical resection of one to three cerebral metastases: results of the EORTC 22952-26001 study. J Clin Oncol.

[45] Yamamoto M, Serizawa T, Shuto T, Akabane A, Higuchi Y, Kawagishi J, Yamanaka K, Sato Y, Jokura H, Yomo S, Nagano O, Kenai H, Moriki A, Suzuki S, Kida Y, Iwai Y, Hayashi M, Onishi H, Gondo M, Sato M, Akimitsu T, Kubo K, Kikuchi Y, Shibasaki T, Goto T, Takanashi M, Mori Y, Takakura K, Saeki N, Kunieda E, Aoyama H, Momoshima S, Tsuchiya K (2014). Stereotactic radiosurgery for patients with multiple brain metastases (JLGK0901): a multi-institutional prospective observational study. Lancet Oncol.

[46] Gaspar L, Scott C, Rotman M, Asbell S, Phillips T, Wasserman T, McKenna WG, Byhardt R (1997). Recursive partitioning analysis (RPA) of prognostic factors in three Radiation Therapy Oncology Group (RTOG) brain metastases trials. Int J Radiat Oncol Biol Phys.

[47] Sperduto PW, Chao ST, Sneed PK, Luo X, Suh J, Roberge D, Bhatt A, Jensen AW, Brown PD, Shih H, Kirkpatrick J, Schwer A, Gaspar LE, Fiveash JB, Chiang V, Knisely J, Sperduto CM, Mehta M (2010). Diagnosis-specific prognostic factors, indexes, and treatment outcomes for patients with newly diagnosed brain metastases: a multi-institutional analysis of 4,259 patients. Int J Radiat Oncol Biol Phys.

[48] Rades D, Dunst J, Schild SE (2008). A new scoring system to predicting the survival of patients treated with whole-brain radiotherapy for brain metastases. Strahlenther Onkol.

[49] Dziggel L, Segedin B, Podvrsnik NH, Oblak I, Schild SE, Rades D (2013). Validation of a survival score for patients treated with whole-brain radiotherapy for brain metastases. Strahlenther Onkol.

[50] Aupérin A, Arriagada R, Pignon JP, Le Péchoux C, Gregor A, Stephens RJ, Kristjansen PE, Johnson BE, Ueoka H, Wagner H, Aisner J (1999). Prophylactic cranial irradiation for patients with small-cell lung cancer in complete remission. Prophylactic cranial irradiation overview collaborative group. N Engl J Med.

[51] Slotman BJ, van Tinteren H, Praag JO, Knegjens JL, El Sharouni SY, Hatton M, Keijser A, Faivre-Finn C, Senan S (2015). Use of thoracic radiotherapy for extensive stage small-cell lung cancer: a phase 3 randomised controlled trial. Lancet..

[52] Warner MH, Beckett GJ (2010). Mechanisms behind the non-thyroidal illness syndrome: an update. J Endocrinol.

[53] Rades D, Bohlen G, Dunst J, Lohynska R, Veninga T, Stalpers L, Schild SE, Dahm-Daphi J (2008). Comparison of short-course versus long-course whole-brain radiotherapy in the treatment of brain metastases. Strahlenther Onkol.

[54] Rades D, Heisterkamp C, Schild SE (2010). Do patients receiving whole-brain radiotherapy for brain metastases from renal cell carcinoma benefit from escalation of the radiation dose?. Int J Radiat Oncol Biol Phys.

[55] Rades D, Panzner A, Dziggel L, Haatanen T, Lohynska R, Schild SE (2012). Dose-escalation of whole-brain radiotherapy for brain metastasis in patients with a favorable survival prognosis. Cancer.

[56] Kyriakakis N, Lynch J, Orme SM, Gerrard G, Hatfield P, Short SC, Loughrey C, Murray RD. Hypothalamic-pituitary axis irradiation dose thresholds for the development of hypopituitarism in adult-onset gliomas. Clin Endocrinol (Oxf). 2019 Mar 15. 10.1111/cen.13971 30873631

[57] Pekic S, Miljic D, Popovic V. Hypopituitarism Following Cranial Radiotherapy. 2018 Oct 1. In: Feingold KR, Anawalt B, Boyce A, et al., editors. Endotext [internet]. South Dartmouth (MA): MDText.com, Inc. 30321012

[58] Sathyapalan T, Dixit S (2012). Radiotherapy-induced hypopituitarism: a review. Expert Rev Anticancer Ther.

[59] Ahmad A. Altered fractionation. In: Perez and Brady’s Principles and Practice of Radiation Oncology, sixth edition 2013.

[60] Littley MD, Shalet SM, Beardwell CG, Ahmed SR, Applegate G, Sutton ML (1989). Hypopituitarism following external radiotherapy for pituitary tumours in adults. Q J Med.

[61] Crowne E, Gleeson H, Benghiat H, Sanghera P, Toogood A (2015). Effect of cancer treatment on hypothalamic-pituitary function. Lancet Diabetes Endocrinol.

[62] Merchant TE, Williams T, Smith JM, Rose SR, Danish RK, Burghen GA, Kun LE, Lustig RH (2002). Preirradiation endocrinopathies in pediatric brain tumor patients determined by dynamic tests of endocrine function. Int J Radiat Oncol Biol Phys.

